# Combined Ultrasound and NaHCO_3_ Treatment Improves Soymilk Stability and Soybean Flour Quality by Regulating the Structure and Properties of Interfacial Soy Proteins

**DOI:** 10.3390/foods15162926

**Published:** 2026-08-20

**Authors:** Lin Zhu, Boyan Jin, Can Li, Zhijun Fan, Qingfeng Ban, Zhongjiang Wang

**Affiliations:** 1 College of Food Science, Northeast Agricultural University, Harbin 150030, China; zhulinnn21@163.com (L.Z.); 17836589001@163.com (B.J.); 15237663035@163.com (C.L.); qfban@neau.edu.cn (Q.B.); 2 Heilongjiang Beidahuang Green and Healthy Food Co., Ltd., Jiamusi 154007, China; 15845177666@139.com

**Keywords:** soybean flour, ultrasound treatment, alkali treatment, soymilk, digestive properties

## Abstract

During soybean processing, insufficient disruption of the soybean cell wall structure limits protein solubilization, thereby reducing the nutritional value and bioavailability of soybean-based products. In this study, ultrasound combined with NaHCO_3_ was used to induce targeted modification of soybean tissue structure and interfacial protein properties, with the aim of improving protein extraction, soymilk stability, and the digestive properties of spray-dried soybean flour. The results showed that single ultrasound treatment and single alkali treatment could increase the protein extraction rate in soymilk. But compared with the untreated control, the combined treatment increased the protein dissolution rate from 70.04% to 81.12% and decreased the protein residue rate in okara to 19.74%. The treatment also reduced the average particle size from 132.60 µm to 90.61 µm, increased the emulsifying activity index to 35.49 m^2^/g. Further experiments showed that the combined treatment of ultrasound and alkali increased the content of interface protein and modified protein conformation. These changes contributed to the improvement of storage stability and ionic stability of soymilk. Furthermore, the resulting spray-dried soybean flour exhibited a more uniform particle distribution with reduced interparticle agglomeration, higher solubility (88.37%), and higher in vitro digestibility (89.48%). Overall, the combined treatment enhanced protein solubility and interfacial behavior, thereby improving soymilk stability and soybean flour quality. These findings provide a useful theoretical basis for the development of soybean flour with improved functional and nutritional properties.

## 1. Introduction

With the increasing attention of consumers to plant-based food and healthy diet, soybean has become one of the most important protein sources in plant-based food because of its high protein content, balanced amino acid composition and good processing characteristics [1]. As a typical soybean-based liquid food, soymilk is not only rich in nutrition, but also has a good market prospect [2]. However, soymilk is not easy to store and transport. In order to extend shelf life and reduce storage and transportation costs, factories usually prepare soymilk into soybean flour products by spray-drying [3]. As a complex heterogeneous dispersion system, the stability of soymilk is closely related to the dissolution rate of protein, the adsorption behavior of protein at the oil–water interface and the interaction between particles [4,5]. Therefore, improving the stability of soymilk and the quality characteristics of spray-dried soybean flour by improving the dissolution rate of soybean protein and changing the structure of soybean protein through physical, chemical and enzymatic methods has become the main line of improving the functionality of soybean flour at home and abroad [6,7]. Dai et al. [8] successfully transformed large insoluble aggregates into water-soluble particles by using a high-energy fluid Microfluidizer combined with alkali-driven treatment, which significantly improved the protein dissolution rate and made the system protein show lower interfacial tension. The prepared emulsion remained stable and free of flocculation after 28 days of storage. Igartúa et al. [9] effectively improved the dissolution rate of protein by pH shift combined with ultrasound and heating treatment, resulting in the reduction in particle size, the change in spatial structure, the improvement of surface hydrophobicity and the significant enhancement of emulsifying ability. Balballi et al. [10] have shown that the extraction of hazelnut protein by alkali treatment can achieve a high protein dissolution rate, but it will cause a large amount of denaturation and aggregation, limiting solubility and digestibility. Studies have shown that a single ultrasound treatment or alkali treatment can regulate protein structure and improve its functional properties to a certain extent, but single treatment often has the problems of limited regulation effect or incomplete structural improvement. In contrast, the synergistic treatment of ultrasound and alkali is expected to have a dual effect of “physical fragmentation chemical regulation”. Although the individual effects of ultrasound or alkali on soy protein have been reported, it remains unclear how the combined treatment regulates soluble protein content and interfacial protein structure, and how these changes are related to both soymilk stability and the physicochemical and digestive properties of spray-dried soybean flour.

Therefore, this study investigated the effects of ultrasound, NaHCO_3_, and the combined treatment on soy proteins in soymilk, with emphasis on the changes in soluble protein content, interfacial behavior, and protein structure, as well as their roles in soymilk stability. In addition, the physicochemical properties and in vitro digestibility of soybean flour prepared by spray-drying were characterized. Previous studies have demonstrated that physical or alkaline treatments can improve specific functional properties of plant proteins, such as solubility, emulsifying properties, and interfacial behavior, through changes in particle size, protein conformation, and aggregation state [6,11,12,13]. However, the relationships among protein structural changes, interfacial behavior, soymilk stability, and the quality attributes of spray-dried soybean flour following combined ultrasound and NaHCO_3_ treatment remain insufficiently understood. Therefore, this study aimed to establish these relationships and to provide insight into the mechanism by which the combined treatment improves product quality.

## 2. Materials and Methods

### 2.1. Materials

The protein extraction rate in soymilk and the protein residual rate in okara were measured following a slightly modified procedure of Cui et al. [14]. In brief, 50 g of intact soybeans was soaked in 0.8% NaHCO_3_ solution at a soybean-to-water ratio of 1:8 (*w*/*v*) for 12 h at 25 °C. The soaking solution was then discarded, and a 1:6 (*w*/*v*) proportion of soybeans to fresh deionized water was used. The soybeans were ground with deionized water in a soymilk maker for 3 min. The resulting soy slurry was subjected to ultrasound treatment using a 6 mm probe at 350 W and 20 kHz for 15 min, with a pulse mode of 2 s on and 2 s off. The sample was maintained in an ice-water bath throughout the treatment to ensure that the temperature remained below 10 °C. Soymilk and okara were collected separately, and the protein extraction rate in soymilk and the protein residual rate in okara were determined. Samples prepared without NaHCO_3_ soaking or ultrasound treatment were defined as the control soymilk/okara. Samples prepared with NaHCO_3_ soaking only were defined as the alkali-treated soymilk/okara. Samples prepared with ultrasound treatment only were defined as the ultrasound-treated soymilk/okara. Samples prepared with both NaHCO_3_ soaking and ultrasound treatment were defined as the ultrasound–alkali-treated soymilk/okara.

### 2.2. Determination of Protein Extraction Rate in Soymilk and Protein Residual Rate in Okara

(1) $$protein\;extraction\;rate\;in\;soymilk(\%)=\frac{m_{1}}{m_{2}}\times 100$$

(2) $$protein\;residual\;rate\;in\;okara(\%)=\frac{m_{3}}{m_{2}}\times 100$$

where m_1_, m_2_, and m_3_ correspond to the protein masses of soymilk, soybeans, and okara, respectively (g). Protein content was established from total nitrogen analysis by the Dumas method [15], using 6.25 as the nitrogen-to-protein conversion coefficient.

### 2.3. Determination of Soymilk Particle Size

Soymilk particle size was analyzed following the procedure of Xue et al. [16]. Soymilk samples were diluted 100 times prior to particle size analysis using a Mastersizer 3000 laser diffraction instrument (Malvern, UK). The refractive index values applied in the analysis were 1.57 for the protein phase and 1.33 for the aqueous phase.

### 2.4. Determination of Soymilk Color

Soymilk color was analyzed using a Chroma Meter (CR-400, Konica Minolta Inc., Osaka, Japan) following the procedure reported by Mehraj Fatema Z. Mulla [17]. The instrument was first calibrated with a blank standard. The probe was then placed on the surface of the soymilk, and the color parameters L*, a*, and b* were measured under standard illuminant C, with a 2° standard observer and diffuse illumination/0° viewing (d/0°) geometry.

### 2.5. Determination of Soymilk Rheological Properties

Rheological measurements of soymilk were analyzed following the procedure of Wang et al. [18]. Rheological measurements were performed using a modular rotational rheometer (HAAKE MARS60, Thermo Fisher Scientific Inc., Waltham, MA, USA) equipped with a 35 mm diameter parallel-plate geometry, with the gap set to 0.3 mm. At 25 °C, apparent viscosity was evaluated within a shear rate range of 0.1–100 s^−1^. Prior to the frequency sweep test, an amplitude sweep was conducted to determine the linear viscoelastic region (LVER). In addition, frequency sweep analysis was conducted from 0.1 to 100 Hz at a constant strain of 0.5% to obtain the storage modulus (G′) and loss modulus (G″).

### 2.6. Determination of Soymilk Emulsifying Properties

Soymilk emulsifying properties were evaluated following the procedure of Zhang et al. [19]. For the determination of emulsifying properties, 30 mL of soymilk obtained under different processing treatments was blended with 10 mL of sunflower seed oil and homogenized at 10,000 rpm for 2 min using a homogenizer (T-25 homogenizer, IKA-Werke GmbH & Co. KG, Staufen, Germany). A 50 μL portion of the freshly prepared emulsion was then added to 5 mL of 0.1% SDS solution and vortex-mixed thoroughly. Absorbance was measured at 500 nm using a UV–Vis spectrophotometer (DU 500, Beckman Coulter, Fullerton, CA, USA) immediately after dilution and recorded as A_0_, followed by a second measurement after the sample had been left to stand for 30 min, recorded as A_30_. EAI and ESI were calculated using the equations below:

(3) $$EAI=\frac{2\times 2.303\times A_{0}\times 100}{C\times L\times 0.25\times 10^{4}}$$

(4) $$ESI=\frac{A_{30}}{A_{0}}\times 100\%$$

In these equations, C and L designate the protein concentration (g/mL) and the optical path length, respectively.

### 2.7. Extraction of Interfacial Proteins

Interfacial proteins were obtained with the procedure outlined by Liao et al. [20]. Briefly, 10% sucrose was added to soymilk subjected to different treatments, followed by homogenization in an ice-water bath (4 °C, 10 min). The samples were then centrifuged at 9056× *g* and 4 °C for 30 min to obtain the cream layer. After dispersion in deionized water, the cream layer was subjected to centrifugation once more under the same parameters to recover the oil bodies. Freshly prepared oil bodies were freeze-dried. Lipids in the oil bodies were extracted with ether. Extraction of oil-body lipids was carried out with a chloroform/methanol mixture (2:1, *v*/*v*). This extraction resulted in separation into a chloroform phase (containing phospholipids), a methanol–water phase, and an interfacial fraction. Interfacial protein isolation was carried out by mixing the interfacial fraction with chloroform/methanol (1:3, *v*/*v*) and water, followed by centrifugation at 9056× *g* for 15 min at 4 °C. The recovered interfacial protein fraction was pre-frozen at −18 °C for 24 h and subsequently lyophilized for 24 h using a freeze dryer.

### 2.8. Determination of Interfacial Protein Content

The interfacial protein concentration was established from total nitrogen analysis by the Dumas method [15], using 6.25 as the nitrogen-to-protein conversion coefficient.

### 2.9. Determination of Interfacial Tension

Interfacial tension was measured following the procedure of Huang et al. [21]. At 25 °C, the droplet profile (5 μL) was continuously recorded using a CCD video camera system with video optical contact angle analysis, and the change in surface tension with adsorption time (t) was monitored over 3200 s.

### 2.10. Determination of Dynamic Interfacial Pressure

The dynamic interfacial pressure was calculated using Equation (5):

(5) $$\pi \;={\;\gamma }_{\mathrm{ow}}-\gamma_{\mathrm{op}}$$

where π is the interfacial pressure, γ_ow_ and γ_op_ denote the oil–water and oil–protein interfacial tensions, respectively.

### 2.11. FTIR Analysis of Soymilk Proteins

FTIR samples were prepared by mixing the protein with KBr at 1:100 (*w*/*w*), followed by grinding and pellet formation following Wei et al. [22]. Spectra were recorded using an FTIR spectrometer (Nicolet 6700, Thermo Fisher Scientific Inc., Waltham, MA, USA) over the range of 500–4000 cm^−1^ at 4 cm^−1^ resolution with 32 accumulated scans.

### 2.12. Circular Dichroism Analysis of Soymilk Proteins

For circular dichroism (CD) analysis, soymilk protein samples were prepared at a concentration of 0.5 mg/mL and scanned from 190 to 260 nm using a CD spectropolarimeter (J1500 CD, JASCO, Tokyo, Japan) following Famouri et al. [23]. For circular dichroism analysis, soymilk protein samples were prepared at 0.5 mg/mL and scanned from 190 to 260 nm with a CD spectropolarimeter. The resulting spectra were subsequently processed using CDNN version 2.1 (Circular Dichroism Neural Network) software to determine secondary structure.

### 2.13. Determination of Particle Size and Zeta Potential of Soymilk Proteins

Following the procedure described by Xue et al. [16], a protein solution (1 mg/mL) was prepared, and the size and surface charge of soymilk protein particles were then measured.

### 2.14. Intrinsic Fluorescence Measurement of Soymilk Proteins

Intrinsic fluorescence spectra of protein solutions (0.1 mg/mL) were recorded using a fluorescence spectrophotometer (RF-6000, Shimadzu Instruments Ltd., Suzhou, China) following Wang et al. [24]. Samples were excited at 290 nm for fluorescence analysis, and the emitted signals were monitored across 300–500 nm with a 5 nm slit width.

### 2.15. Determination of Surface Hydrophobicity of Soymilk Proteins

Based on the procedure of Yao et al. [25], a protein solution with a concentration of 0.1 mg/mL was obtained. A 4 mL aliquot of soymilk was mixed with 40 μL of 8-anilino-1-naphthalenesulfonic acid solution (8 mmol/L, pH 7.4) and incubated in the dark for 2 h. Fluorescence intensity was then recorded on a fluorescence spectrophotometer (RF-6000, Shimadzu Instruments Ltd., Suzhou, China) at 390 nm for excitation and 470 nm for emission.

### 2.16. Macroscopic Stability of Soymilk

Different treated soymilk samples were stored in glass bottles at 25 °C. Macroscopic stability was evaluated by observing phase separation over 0–5 days.

### 2.17. Determination of Ionic Stability of Soymilk

Different treated soymilk samples were stored in glass bottles at 25 °C after adding 0.5% NaCl. Ionic stability was evaluated by observing phase separation over 0–5 days.

### 2.18. Preparation of Soybean Flour

Soybean flour samples were prepared by spray-drying soymilk obtained from four different pretreatments: untreated, alkali treatment, ultrasound treatment, and combined ultrasound and alkali treatment. The spray-drying system was set with an inlet temperature of 150 °C and an outlet temperature of 90 °C. After the operating parameters became stable, the soymilk samples were pumped into the atomizer at a constant flow rate of 300 mL/h using a peristaltic pump for drying. The dried soybean flour samples were quickly collected in sealed containers and stored in a desiccator after cooling to 25 °C.

### 2.19. Determination of Soybean Flour Solubility

With reference to Guo et al. [26], soybean flour solubility was assessed as follows. A 0.5 g portion of soybean flour was dispersed in 100 mL of distilled water and magnetically stirred at 500 rpm for 2 h to ensure complete dispersion. The prepared dispersion was centrifuged at 1006× *g* for 5 min, after which 25 mL of supernatant was collected and dried at 100 °C until the sample reached a constant weight. The solubility value was obtained from the ratio of the dry matter recovered from the supernatant to the total dry matter of the dispersion.

### 2.20. Determination of Microstructure of Soybean Flour 

After freeze-drying, the microstructure of soybean flour samples subjected to different treatments was examined using a tungsten filament scanning electron microscope (SEM; S-3400N, Hitachi High-Technologies Corp., Tokyo, Japan) according to Zhang et al. [27]. An appropriate amount of each sample was uniformly distributed on conductive adhesive tape, secured, and then observed at an accelerating voltage of 5 kV. Images were obtained at 1500×.

### 2.21. In Vitro Digestion of Soybean Flour

In vitro digestion was carried out as described by Luo et al. [28]. In brief, 200 mg soybean flour was first mixed with 10 mL of water and shaken to wet the sample. Then, 10 mL of simulated gastric fluid (containing 0.32 g pepsin) was added, and then brought to pH 2. The mixture was incubated in a constant-temperature shaker at 100 r/min for 2 h. Subsequently, 10 mL of simulated intestinal fluid (containing 0.1 g of trypsin, 0.2 g of bile salts, and 0.832 g of calcium chloride) was added, and then brought to pH 7. Shaking was continued for another 2 h. After digestion, the reaction vessel was immediately placed in an ice bath for 10–15 min to rapidly reduce enzyme activity and terminate the digestion. After cooling to 4 °C, the samples were used for further analysis.

### 2.22. Determination of Soybean Flour Digestibility

Soybean flour digestibility was evaluated following the procedure of Cahill et al. [29]. Under cooling conditions at 4 °C, the digest was centrifuged for 20 min at 12,000× *g*. The supernatant was then recovered, while the sample was resolved into soluble and insoluble fractions. The protein content of the supernatant was analyzed by the Dumas combustion method, and digestibility was calculated using Equation (6):

(6) $$Soy\;powder\;digestibility\;=\;\frac{M_{1}}{M_{2}}\;\times \;100\%$$

where M_1_ represents the protein content in the supernatant after digestion, whereas M_2_ denotes the protein content of the sample prior to digestion.

### 2.23. SDS-PAGE Analysis of Soybean Flour After In Vitro Digestion

SDS-PAGE analysis was carried out with slight modifications based on the procedure reported by Xing et al. [30]. Soy residue protein and soymilk protein were prepared as 1 mg/mL solutions. For electrophoretic analysis, each sample (40 μL) was mixed with loading buffer (10 μL) and denatured at 100 °C for 5 min. An aliquot of 8 μL was then loaded onto the gel. Electrophoresis proceeded at 80 V during the first 60 min and at 120 V during the subsequent 60 min. Once the run was completed, the gel was immersed in Coomassie Brilliant Blue for 1 h and subsequently washed with deionized water. The resulting bands were documented and evaluated using Image Lab software version 4.0.

### 2.24. Statistical Analysis

Triplicate determinations were made for all samples, and the resulting data are given as mean ± standard deviation. Group comparisons were based on a one-way ANOVA, with Duncan’s multiple range procedure applied to separate significant differences at the 0.05 level. Origin 2021 was used to process the statistical results.

## 3. Results

### 3.1. Protein Extraction Yield of Soymilk and Residual Protein Content of Okara

**Figure 1:**
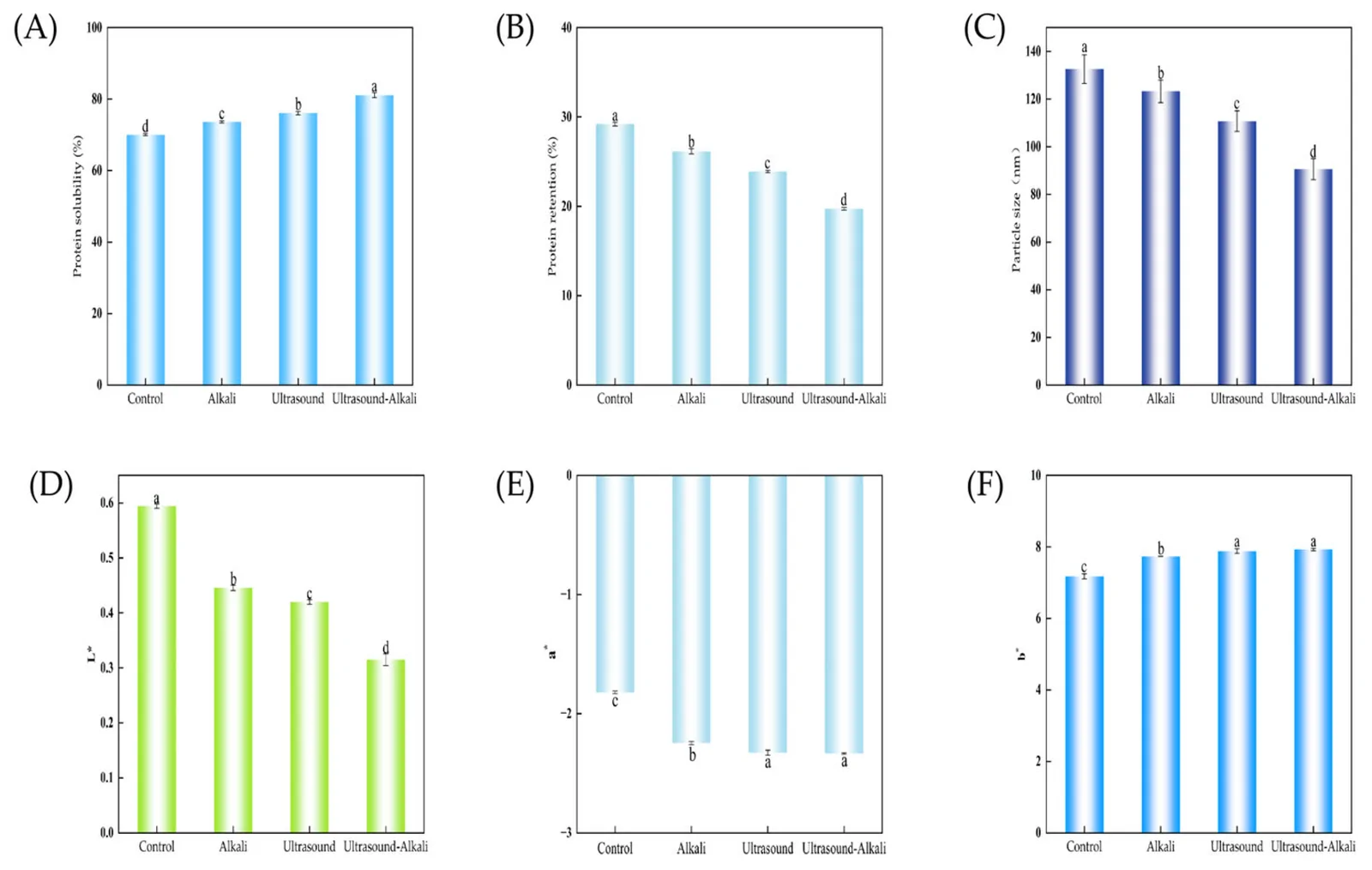
Protein dissolution rate (**A**), protein residue rate in okara (**B**), particle size (**C**) and color parameters, including L* (**D**), a* (**E**), and b* (**F**), of soymilk under different treatments. Different letters indicate significant differences (*p* < 0.05).

Protein dissolution rate is the core index to evaluate the extraction efficiency of soybean protein [31]. The higher its content, the richer the nutrition. As illustrated in Figure 1A, the protein dissolution rate of soymilk with different processing treatments showed an upward trend. The protein dissolution rate of soymilk without any treatment was the lowest, which was 70.04%. The content of soluble protein in soymilk treated by ultrasound combined with alkali was the highest, which was 81.12% (*p* < 0.05). This result indicates that the combined treatment effectively promoted protein dissolution and transfer into the soymilk. On the one hand, under alkaline conditions, protein molecules are negatively charged and intermolecular electrostatic repulsion is enhanced. This will promote the dissociation of protein aggregates into smaller soluble units, thus increasing their dispersion in the aqueous phase [32]. On the other hand, the micro jet generated by ultrasound destroys the plant cell wall, promotes the release of substances in the cell, and enhances the mass transfer efficiency [33].

As depicted in Figure 1B, the protein residue rate of soybean dregs with different processing treatments showed a downward trend. The residual rate of protein in soybean dregs after combined ultrasound and alkali treatment was the lowest, at 19.74%, indicating that more proteins were transferred into the soymilk during the combined treatment, thereby improving the protein nutritional content of the soymilk.

### 3.2. Particle Size of Soymilk

The particle size directly affects the taste and stability of soymilk protein and other nutrients [34]. As illustrated in Figure 1C, the particle size of soymilk in different processing treatments showed a downward trend. Untreated soymilk showed a mean particle size of 132.6 µm, whereas that of the combined treatment decreased to 90.61 µm. The combined treatment significantly inhibited the aggregation of soymilk. This may be related to the destruction of the interaction between proteins. Due to the increase in soluble protein, a protective film is formed on the surface of soymilk to prevent droplet aggregation, making the droplet distribution more uniform, thus reducing the turbidity and improving the quality of soymilk [35]. Meanwhile, a smaller particle size promotes the formation of soluble proteins, which is supported by the observed increase in soluble protein content [36].

### 3.3. Color of Soymilk

In the determination of the color of soymilk, the brightness value L*, the red–green degree a*, and the yellow–blue degree b* are commonly used to express color [37]. Figure 1D presents that the L* value of soymilk without any treatment was the highest, which was 0.59 (*p* < 0.05), and the L* value of soymilk treated by ultrasound combined with alkali was the lowest, which was 0.32. The results showed that the soybean flour prepared by ultrasound synergistic alkali treatment was darker. This may have been due to the higher content of soluble protein in soymilk treated by ultrasound combined with alkali, which led to a reduction in light-scattering intensity and poor light transmittance, thus resulting in a lower brightness value. In addition, alkali treatment may have promoted the dissolution of flavonoids, polyphenols, and other colored substances in soybeans, as well as the Maillard reaction caused by the local high temperature generated by ultrasound cavitation, resulting in the formation of brown substances and the darkening in color [38]. As shown in Figure 1E,F, the smaller a* value and larger b* value indicated that, compared with control soymilk, the red color of soymilk treated by ultrasound combined with alkali was less obvious, but the yellow color was more obvious. This may have been due to the combination of the increased soluble protein and pigment molecules to form a soluble complex, leading to the color change in soymilk.

### 3.4. Rheological Properties of Soymilk

**Figure 2:**
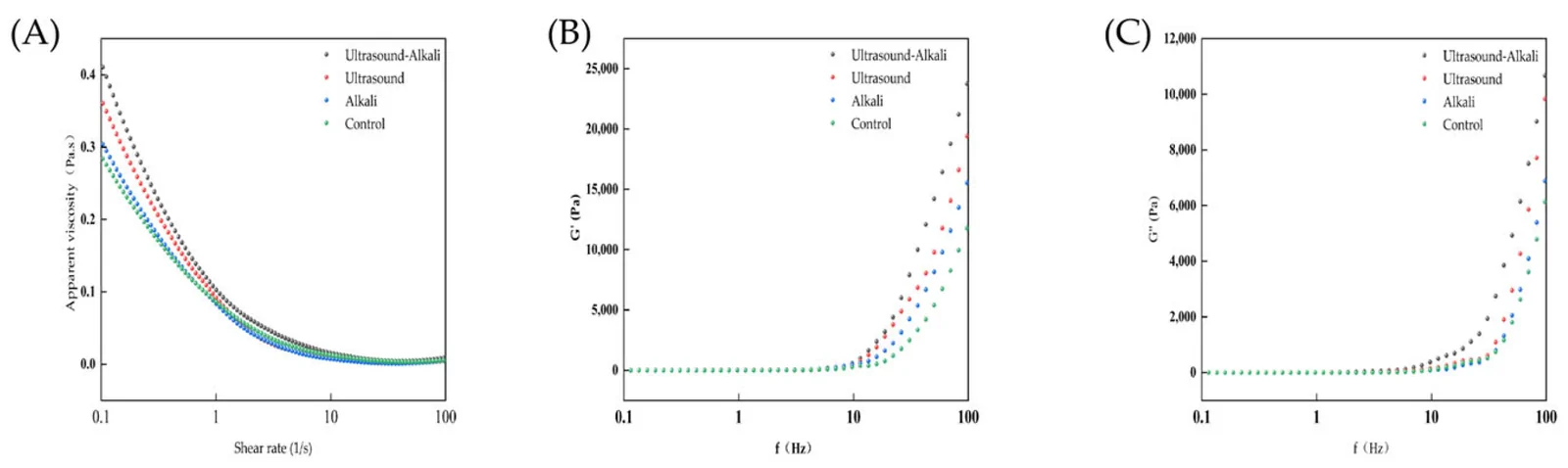
Apparent viscosity (**A**), storage modulus (G′) (**B**) and loss modulus (G″) (**C**) of soymilk under different treatments.

Apparent viscosity is closely related to the intermolecular interactions and flow properties of a liquid, and therefore serves as an indirect parameter for evaluating oral perception and the textural characteristics of soymilk [39]. As presented in Figure 2A, with the increase in shear rate, the apparent viscosity of four kinds of soymilk decreased and stabilized, showing the shear thinning behavior unique to pseudoplastic fluid. This may be due to the structural damage of soymilk during high-speed shear. The viscosity of soymilk treated by ultrasound and alkali is the highest, while that of control soymilk is the lowest. Generally, with the increase in viscosity, oil droplets were prevented from moving freely, aggregating, and undergoing phase separation, making soymilk more stable. Huang et al. [40] reported that higher protein levels led to smaller oil droplets, which resulted in a denser and more compact droplet arrangement. The increased interfacial contact between the smaller droplets, along with the higher Laplace pressure, enhanced their resistance to shear-induced deformation, thereby increasing the apparent viscosity.

In rheological analysis, G′ is regarded as an indicator of the energy stored elastically by the material, whereas G″ reflects the energy dissipated through viscous flow. As illustrated in Figure 2B,C, with the increase in frequency, the elastic modulus and viscosity modulus of soymilk treated with four different treatments continue to increase. The elastic modulus of soymilk treated by ultrasound combined with alkali was the highest, and that of control soymilk was the lowest (*p* < 0.05). This indicates that the soymilk after combined treatment has a more stable three-dimensional network structure [41]. This may be explained by the increase in soluble protein content, which improves the elastic modulus of soymilk, and then enhances the anti-stratification stability of the emulsion. In addition, ultrasound combined with alkali treatment led to smaller soymilk particles and dense droplet arrangement, which significantly enhanced the elastic modulus of soymilk [42]. The elastic modulus of soymilk treated by ultrasound combined with alkali was higher than that of viscosity, indicating that the elastic response was dominant.

### 3.5. Emulsifying Activity of Soymilk

**Figure 3:**
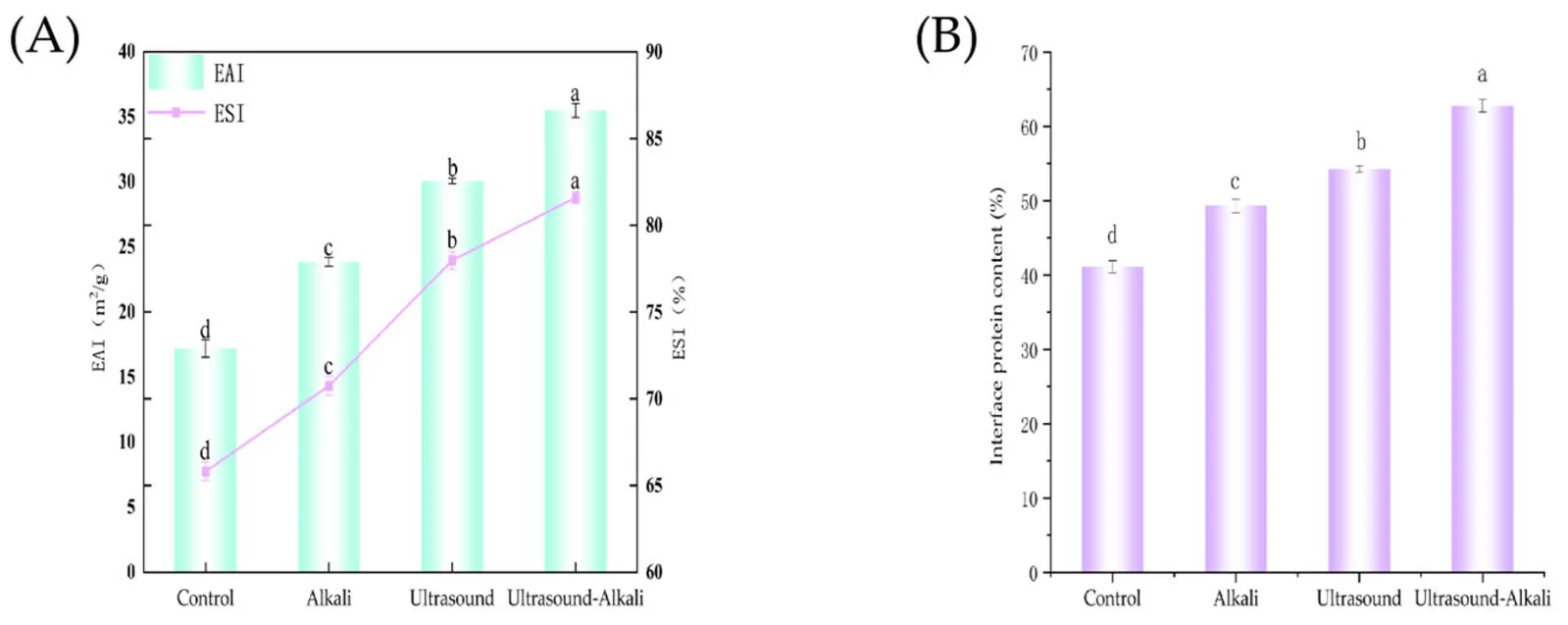
Emulsifying activity (**A**) and interfacial protein content (**B**) of soymilk under different treatments. Different letters indicate significant differences (*p* < 0.05).

EAI and ESI are commonly used to evaluate the emulsifying and emulsion-stabilizing properties of plant proteins [43]. EAI and ESI of soymilk are shown in Figure 3A. The results showed that the EAI of soymilk with different processing treatments showed an upward trend. The EAI of common soymilk was the smallest, which was 17.20 m^2^/g. The EAI of alkali treated and ultrasound treated soymilk was 23.85 m^2^/g and 30.07 m^2^/g, respectively. The EAI of soymilk treated by ultrasound combined with alkali was the largest, which was 35.49 m^2^/g (*p* < 0.05). The ESI of soymilk obtained by different treatments showed a similar trend to that of EAI. This may be due to the structural changes in protein in soymilk caused by the combined treatment, exposing more hydrophobic groups and flexible regions [44]. Therefore, proteins with highly flexible structures can quickly respond to the oil–water interface and reduce the adsorption energy barrier through conformational adjustment. This behavior can form a thicker and more viscoelastic interface layer and improve the emulsifying property of soymilk [45].

### 3.6. Interfacial Protein Content

The interfacial protein content reflects the proportion of protein adsorbed at the oil–water interface. It is widely considered an important indicator of the interfacial adsorption ability of proteins. More small-sized protein molecules migrate to the interface and remain adsorbed there, which is generally beneficial for the formation of a stronger and more complete interfacial layer [46]. Figure 3B shows that different processing treatments generally enhanced the interfacial protein content of soymilk. In particular, the combined-treatment sample reached 62.77%, which was the highest among all groups, whereas control soymilk remained at the lowest level (41.14%). Different processing treatments increased the protein content of soymilk, with the combined ultrasound and alkali treatment resulting in a particularly significant increase (*p* < 0.05). As the extracted protein content increased, the interfacial protein content increased accordingly. This may be attributed to the higher level of extracted proteins, which enhanced the interfacial adsorption kinetics and increased the interfacial protein coverage density. This is consistent with the findings of Chen et al. [47], who explored the emulsifying stability and interfacial adsorption behavior of pea protein and found that an increase in pea protein content led to a higher interfacial protein content. This may be because a higher protein concentration allows more proteins to be adsorbed at the O/W interface.

### 3.7. Interfacial Tension

**Figure 4:**
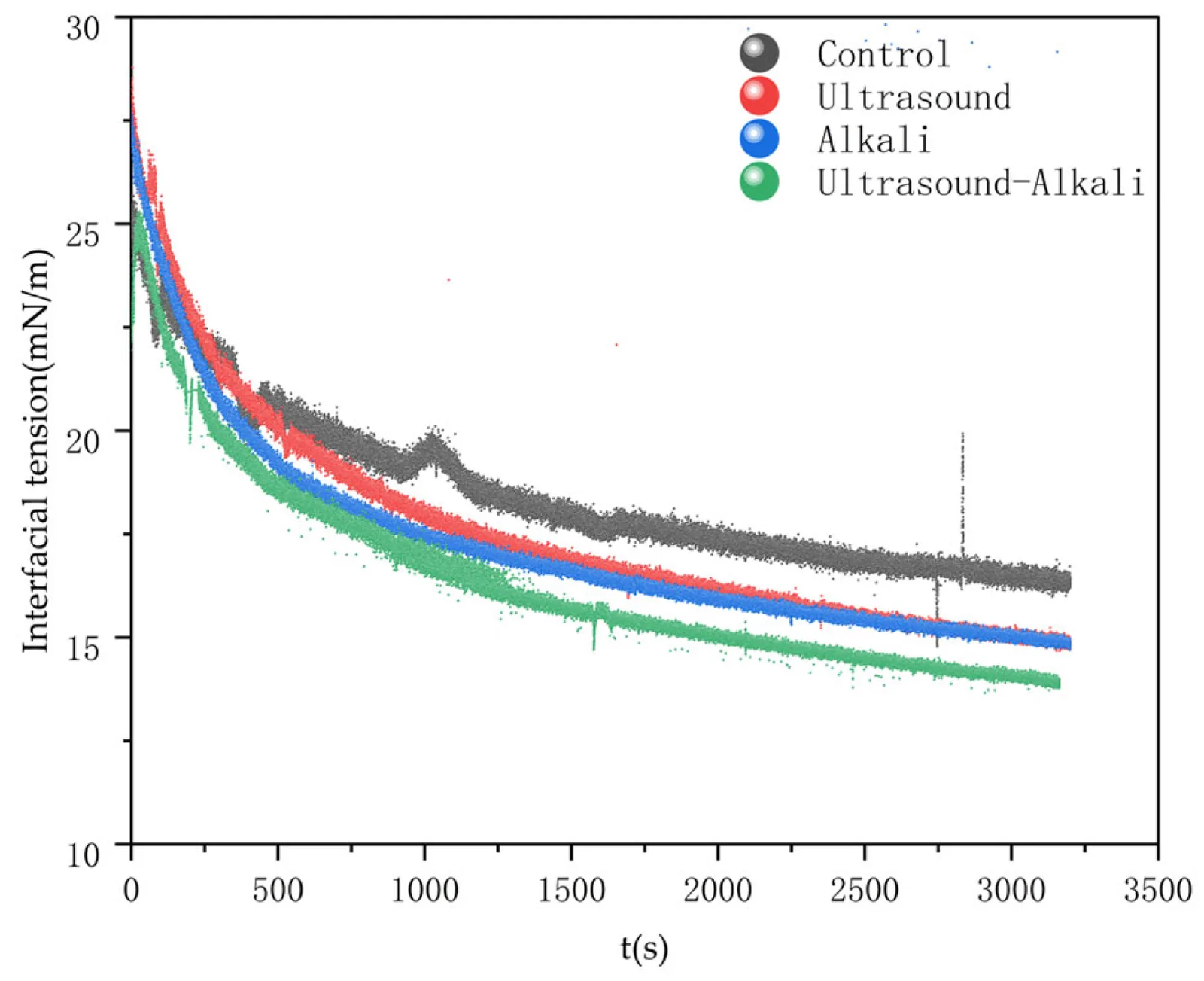
Interfacial tension of soymilk under different treatments.

Interfacial tension is an important parameter for evaluating the interfacial properties of proteins. The decrease in interfacial tension means that the stability and anti-destructive ability of emulsion are enhanced [48]. As shown in Figure 4, the interfacial tension of soymilk interfacial proteins in different processing treatments decreased with time and gradually tended to balance (*p* < 0.05). For all protein samples, the adsorption behavior was characterized by an initial sharp decrease in interfacial tension due to rapid diffusion and adsorption, and then by a slower plateau phase associated with molecular rearrangement at the interface. The gradual decrease in interfacial tension indicates that the interfacial protein molecules are successfully adsorbed at the oil–water interface [49]. At equilibrium, the interfacial protein content decreased in the following order: control soymilk > alkali-treated soymilk > ultrasound-treated soymilk > soymilk treated with combined ultrasound and alkali. The increase in soluble protein content led to greater interfacial coverage by protein molecules, which effectively reduced the interfacial free energy and consequently decreased the interfacial tension. When the interfacial protein reached saturation adsorption, the interfacial tension decreased to the minimum and tended to be stable [50]. In addition, ultrasound combined with alkali treatment leads to the protein structure, and the interface protein tends to be flexible structure. The enhanced flexibility promotes the hydrophilic and hydrophobic balance of interface proteins, thus reducing the energy barrier of protein adsorption to the oil–water interface. Lian Ziteng et al. [51] demonstrated that the increased flexibility of soy protein promoted its effective adsorption at the interface, as reflected by a decrease in interfacial tension.

### 3.8. Dynamic Interfacial Pressure

**Figure 5:**
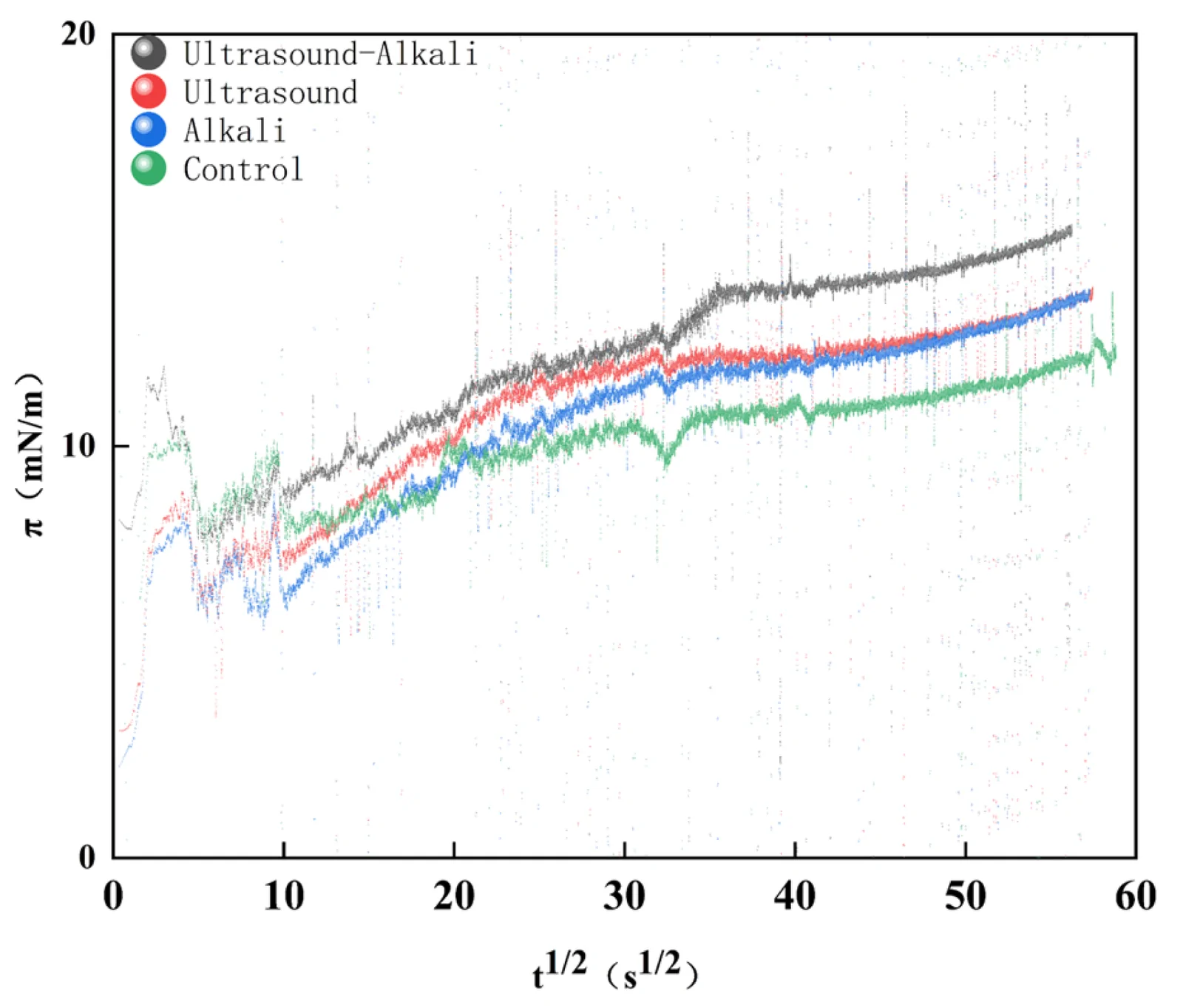
Dynamic interfacial pressure of soymilk under different treatments.

By monitoring the variation in interfacial tension (γ_ow_, γ_op_) with time (t), dynamic interfacial pressure can be obtained and used to evaluate protein adsorption capacity [52]. Figure 5 provides that the interfacial proteins (π) from the four soymilk samples gradually increased with adsorption time, indicating that they could spontaneously adsorb the oil/water interface. Subsequently, the proteins began to diffuse to and penetrate the interface, and finally rearranged to form a viscoelastic interfacial film, thereby stabilizing the soymilk emulsion. At the beginning of adsorption, protein hydrophobic groups quickly reached the oil–water interface. They then spread along the interface, leading to a marked rise in interfacial pressure in all samples [53]. Following this fast adsorption phase, the adsorption rate declined, and the interfacial pressure rose more slowly. This may be attributed to the adsorption behavior of proteins at the oil/water interface. When the interfacial pressure reached equilibrium, the interfacial proteins of soymilk treated with combined ultrasound and alkali exhibited the highest interfacial pressure value (*p* < 0.05), indicating stronger surface activity. This may result from the greater accumulation of proteins at the interface. More protein molecules are adsorbed on the interface through conformational rearrangement, forming a dense interface film, which increases the interface pressure [54]. In addition, combined ultrasound and alkali treatment altered the intermolecular interactions among proteins and enhanced the structural flexibility of interfacial proteins. This further promoted molecular cross-linking and the formation of small soluble aggregates. These structural changes raised the interfacial pressure and consequently improved protein adsorption at the oil/water interface [55].

### 3.9. Fourier Transform Infrared Spectroscopy of Soymilk Proteins

**Figure 6:**
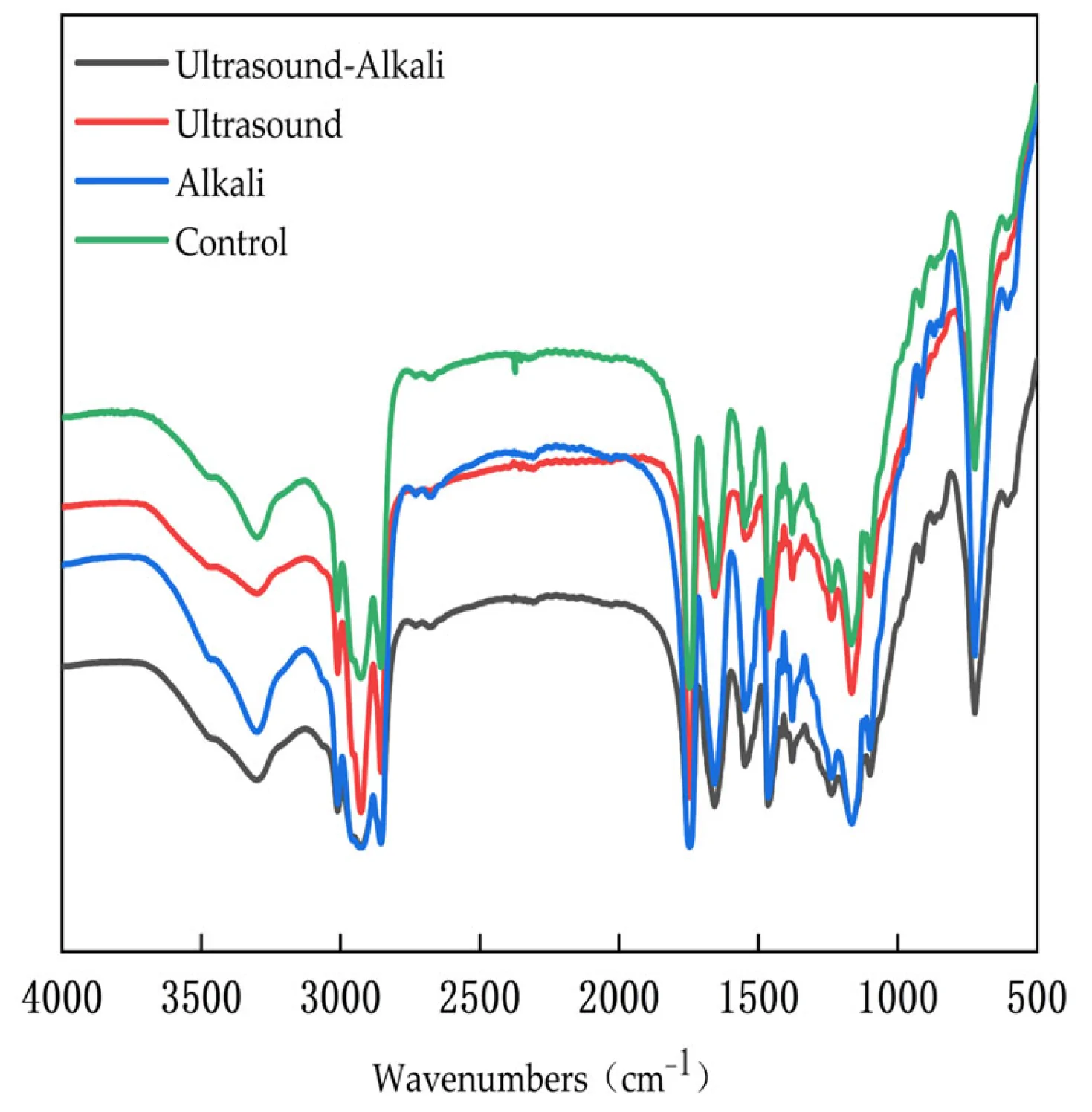
Fourier Transform Infrared Spectroscopy of soymilk under different treatments.

FTIR can effectively recognize the changes in protein secondary structure, and detect intermolecular hydrogen bonds and electrostatic interactions. The four strong bands observed are attributed to amide A (3250–3300 cm ^−1^), amide B (2800–3000 cm ^−1^), amide I (1700–1600 cm ^−1^) and amide II (1600–1500 cm ^−1)^. They correspond to the characteristic absorption peaks of oxygen hydrogen bond, carbon hydrogen bond, carbon=oxygen bond and nitrogen hydrogen bending, respectively [56]. The Fourier transform infrared spectra of soymilk proteins after different treatments are shown in Figure 6. All samples show similar characteristic absorption peaks. This indicates that combined treatment can alter the chain structure and functional group distribution of proteins without changing the types of functional groups [57]. In the amide A region, the ultrasound–alkali treatment induced a slight shift in the band toward a lower wavenumber (red shift) compared with the control. This red shift suggests enhanced or rearranged hydrogen-bonding interactions involving protein N–H groups and associated water molecules, which may be related to the exposure of polar groups and enhanced hydration after protein conformational relaxation. Compared with the single treatment, the change in combined treatment was more obvious, indicating that it can promote the looseness of protein conformation and improve the degree of hydration [58]. Moreover, changes in the peak shape and relative intensity of the amide I/II regions suggested alterations in the hydrogen-bonding environment and protein conformation after treatment. These variations may be related to aggregate dissociation, partial unfolding of proteins, and rearrangement of the secondary structure [59].

### 3.10. Secondary Structure of Soymilk Proteins

The secondary structure of soymilk protein was analyzed by circular dichroism. Changes in the secondary structure composition resulting from the various treatments are listed in Table 1. In the untreated group, the contents of α-helix, β-sheet, β-turn, and random coil were 25.53%, 12.53%, 20.43%, and 41.57%, respectively. Following alkali treatment, ultrasound treatment, and combined ultrasound–alkali treatment, the α-helix content decreased, whereas the contents of β-sheet, β-turn, and random coil generally increased. The treatment exposes more polar groups, such as amide, carboxyl and amino groups, to the water environment. The hydrated layer on the protein surface is thus thickened. Water molecules and polar groups form a hydrogen bond network, which increases the degree of freedom of motion of molecular fragments. This enhanced hydration weakens the rigid interaction between molecules to a certain extent, thus improving the structural flexibility of interface proteins [60]. In particular, after combined ultrasound–alkali treatment, the α-helix content decreased from 25.53% in the untreated group to 18.20%, whereas the contents of β-sheet, β-turn, and random coil increased from 12.53%, 20.43%, and 41.57% to 15.37%, 24.77%, and 44.23%, respectively. These results suggest that combined ultrasound and alkali treatment disrupts the intermolecular and intramolecular interactions that maintain the compact protein conformation, such as hydrophobic interactions, hydrogen bonds, and disulfide bonds, thereby inducing a transition from a rigid, ordered state to a flexible, disordered one. This change facilitates the rapid adsorption of proteins at the oil–water interface and the formation of a dense interfacial film.

### 3.11. Particle Size and Zeta Potential of Soymilk Proteins

**Figure 7:**
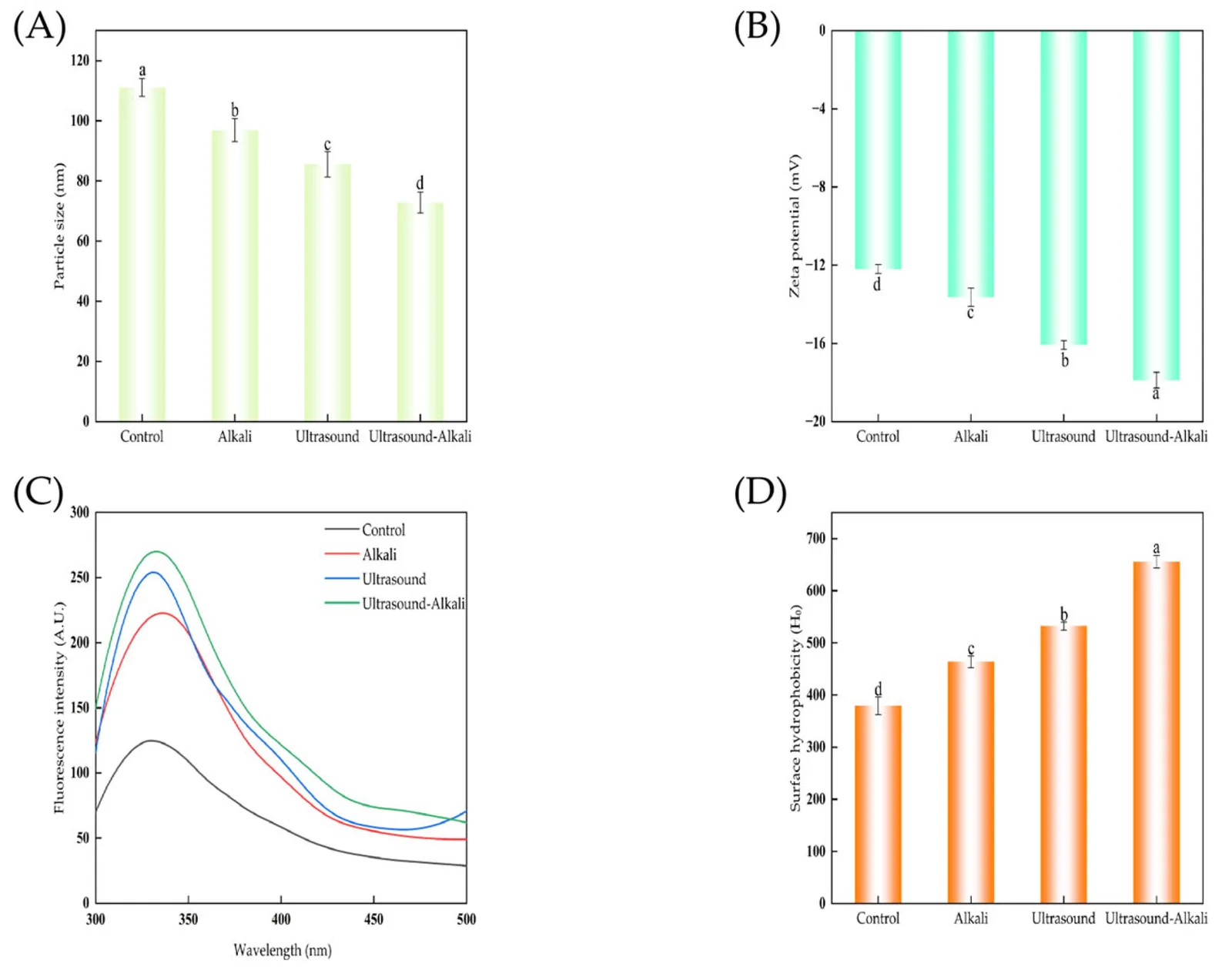
Particle size (**A**), zeta potential (**B**), intrinsic fluorescence spectra (**C**) and surface hydrophobicity (**D**) of soymilk under different treatments. Different letters indicate significant differences (*p* < 0.05).

Particle size reflects the distribution and aggregation of proteins, and has an important impact on the functional properties of proteins. Figure 7A shows that, compared with the untreated group, different processing treatments reduced the particle size of interfacial proteins. This may be because, in the untreated group, most proteins formed insoluble aggregates due to strong intermolecular interactions and existed in a tightly aggregated state. After ultrasound treatment further dispersed the particles and reduced aggregation, thereby significantly decreasing the particle size of interfacial proteins. Compared with alkali treatment alone or ultrasound treatment alone, combined ultrasound and alkali treatment resulted in a greater reduction in the particle size of interfacial proteins, which reached 111.13 nm (*p* < 0.05). As Lian et al. [61] reported, increased protein flexibility enhanced protein–solvent interactions, which thinned the effective hydration layer and further reduced the effective particle size.

Zeta potential reflects the net charge on the surface of particles and is an important index to evaluate the aggregation and stability of particles in solution. Figure 7B illustrates that the absolute value of zeta potential of soymilk protein with different processing treatments showed an upward trend. The absolute value of protein potential in different treatment groups was higher than that in the untreated group. This is because alkali treatment makes the carboxyl group in protein molecules more easily deprotonated, leading to the increase in negative surface charge [62].

### 3.12. Intrinsic Fluorescence Spectra of Soymilk Proteins

Intrinsic fluorescence spectra can reflect the fluorescence characteristics of specific amino acid residues, especially tryptophan, tyrosine and phenylalanine, as well as the changes in protein conformation [63]. Figure 7C shows the intrinsic fluorescence spectra of interfacial proteins in soymilk subjected to different processing treatments. Compared with the untreated group, the fluorescence intensity of the interfacial proteins increased after different processing treatments. This may be attributed to the unfolding of the interfacial protein structure induced by processing. When proteins change from a compact globular structure to a looser conformation, tryptophan residues originally buried inside the molecule become exposed to a polar environment, thereby increasing the fluorescence intensity. This is similar to the results reported by Carvalho et al. [64], indicating that protein unfolding exposed more tryptophan and tyrosine residues and thus enhanced the fluorescence intensity. Compared with alkaline treatment alone or ultrasound treatment alone, the interfacial proteins treated by ultrasound combined with alkali treatment was the highest fluorescence intensity, reaching 111.13 nm (*p* < 0.05). This suggests that combined ultrasound and alkali treatment induced a greater degree of unfolding of the interfacial protein structure, resulting in greater exposure of fluorescent chromophores [59]. In addition, compared with the interfacial proteins of untreated soymilk, the maximum fluorescence emission peak of the interfacial proteins after combined ultrasound and alkali treatment showed a red shift. This indicates that combined treatment altered the tertiary structure of soymilk proteins and caused tryptophan residues to move toward a more hydrophilic microenvironment [65].

### 3.13. Surface Hydrophobicity of Soymilk Proteins

As amphiphilic biomacromolecules, the degree of exposure of hydrophobic groups of proteins directly determines the adsorption capacity at the oil–water interface [66]. Figure 7D presents the surface hydrophobicity values of soymilk interface proteins with different processing treatments. The surface hydrophobicity of soymilk interface protein with different processing treatments showed an upward trend. Compared with the untreated group, the surface hydrophobicity of interfacial proteins increased after different treatments. This may be attributed to the loosening of the protein structure induced by alkali treatment alone, which exposed amino acid residues originally buried within the molecules to the molecular surface, thereby increasing surface hydrophobicity [67]. In addition, the strong mechanical shear generated by ultrasound further disrupted the aggregated structure of the proteins, promoting the dissociation and stretching of molecular chains and exposing more hydrophobic region [44]. Among all treatments, the interfacial protein subjected to combined ultrasound and alkali treatment exhibited the highest surface hydrophobicity (655.71), which was significantly higher than that of the samples treated with alkali alone or ultrasound alone (*p* < 0.05). It shows that the synergistic effect of the two is more effective than a single treatment, which can promote protein molecules to unfold to a greater extent, expose more hydrophobic sites, and improve the surface hydrophobicity, which is consistent with previous studies.

### 3.14. Storage Stability of Soymilk

**Figure 8:**
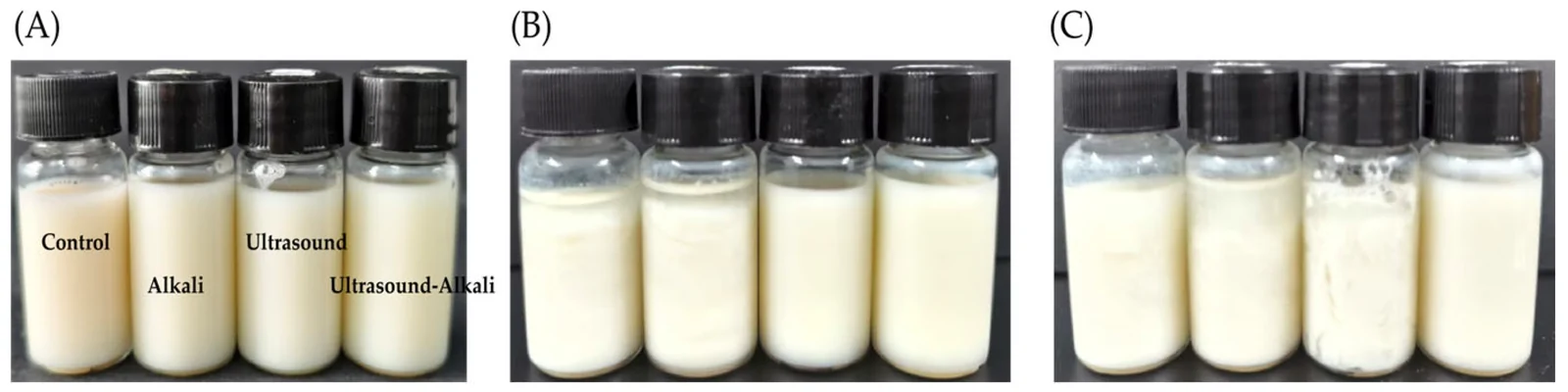
Effect of different processing treatments on the storage stability of soymilk: storage for 1 (**A**), 3 (**B**), and 5 days (**C**).

Stability is an important index to measure the functional properties and storage quality of soymilk. The storage stability results of soymilk with different processing treatments are shown in Figure 8. After storage for one day, the appearance of the samples in each group was relatively uniform, and no obvious stratification was observed; after storage for three days, the control soymilk appeared delamination, indicating that the emulsion system began to lose stability. This phenomenon may be related to the low dissolution rate of common soymilk protein: the lack of adsorbable protein at the interface leads to incomplete coverage of the oil–water interface, and it is difficult to form a continuous dense interface film, which is more prone to flocculation, floatation and macro stratification. In contrast, soymilk subjected to combined ultrasound and alkali treatment still maintained good uniformity after three days, indicating that the combined treatment significantly delayed the instability process of the system. This can be attributed to the high content of soluble proteins. This agrees with the study of Chen Hai et al. [68], who showed that as the protein concentration increased, proteins were more readily adsorbed at the droplet interface, forming a dense interfacial layer that effectively prevented droplet aggregation. After storage for five days, the structure of control soymilk, ultrasound treated soymilk and alkali treated soymilk were damaged to varying degrees, while the ultrasound combined with alkali treatment group still showed good stability. The reason may be that the synergistic treatment makes the particle size of the system smaller and more evenly distributed: smaller particles are easier to migrate and adsorb to the oil–water interface, forming a more fully covered and evenly distributed interface protein layer [69]. This interface film can effectively resist the instability mechanisms such as droplet coalescence and Ostwald ripening, thus significantly improving the storage stability of soymilk.

### 3.15. Ionic Stability of Soymilk

**Figure 9:**
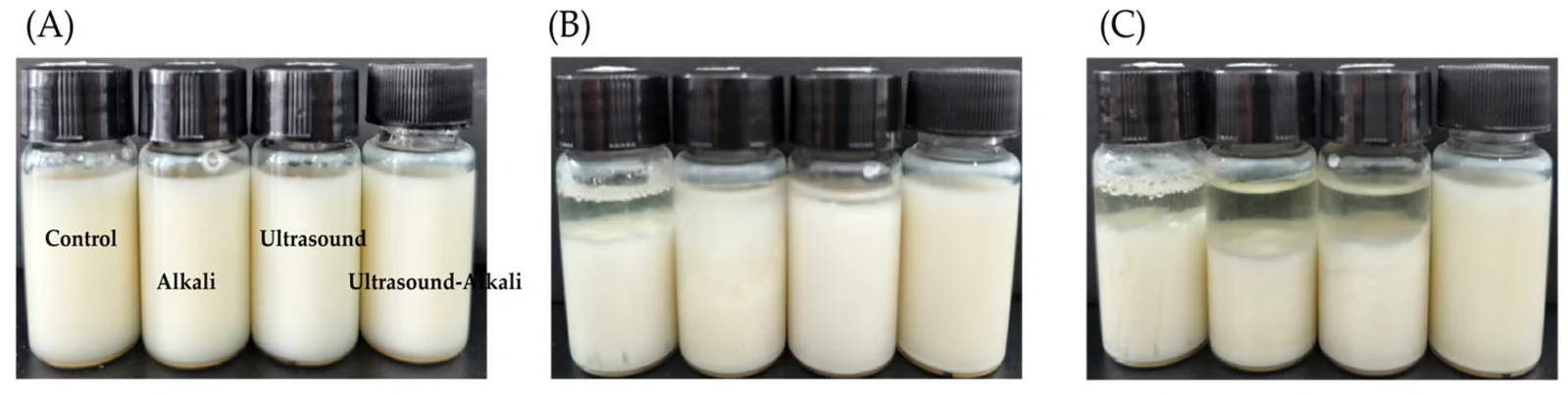
Effect of different processing treatments on the ionic stability of soymilk: storage for 1 (**A**), 3 (**B**), and 5 days (**C**).

Soymilk is a protein fat colloid system, and salt and calcium magnesium plasma will shield the charge, bridge and promote flocculation and precipitation stratification; ion stability can be used to evaluate salt resistance, mineral strengthening resistance and shelf stability [70]. The ionic stability of soymilk processed in different ways is presented in Figure 9. Adding NaCl to soymilk usually affects the stability of soymilk, which may be due to the ability of salt ions to regulate various electrostatic interactions in the emulsion, including attraction and repulsion [58]. The addition of salt tends to shield the electrostatic interaction between droplets, resulting in the reduction in electrostatic repulsion between droplets, which leads to flocculation. When the NaCl concentration was 0.5%, it was observed that on the third day, the control soymilk began to appear stratification, and the appearance of other soymilk was good. This may be due to the difference in the content of soluble protein in soymilk, which leads to the difference in the content of interface protein, and the content of interface protein is the key reason to determine the thickness of interface layer. The thicker interface adsorption layer can better resist the influence of salt ions [71]. However, on the fifth day, the stratification phenomenon of control soymilk, alkali treated soymilk and ultrasound soymilk was observed to varying degrees, while the stratification phenomenon of ultrasound and alkali treated soymilk was not observed, showing good stability. This is likely associated with the increase in protein flexibility by ultrasound combined with alkali treatment, forming a thicker steric barrier layer and electrostatic shielding layer, which has a certain buffer capacity for ions in the solution [72].

### 3.16. Microstructure of Soybean Flour

**Figure 10:**
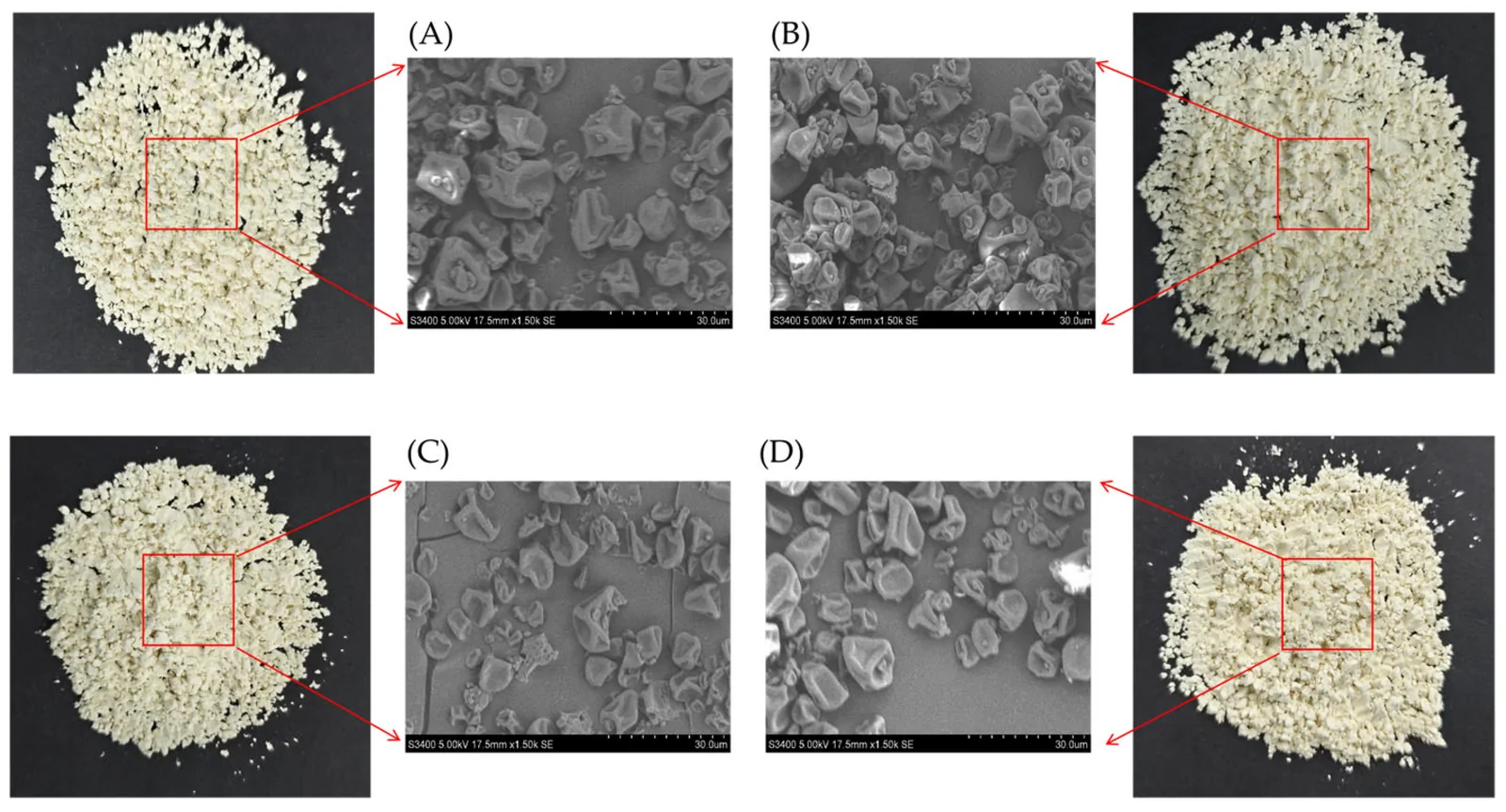
Macroscopic and microscopic images of differently processed soybean flour: control (**A**); alkali (**B**); ultrasound (**C**); ultrasound–alkali (**D**).

Scanning electron microscope was used to observe the micro morphology of soybean flour treated with different methods. The results are presented in Figure 10. There was some heterogeneity in the particles of each group, most of which showed contraction or collapse, and only a few remained relatively complete spherical. Compared with the untreated group, the particle size of soybean flour treated by ultrasound and alkali was smaller, the distribution was more dispersed, and the agglomeration phenomenon between particles was weakened [73]. In the untreated group, the particles were larger and dense. This change may be related to the improvement of soymilk dispersion and system stability [74]. Ultrasound combined with alkali treatment can refine the fat globules and protein aggregates, improve the uniformity of feed and liquid, which is conducive to atomization and formation of smaller particle size and more uniform distribution of powder particles [32]. It shows that the processing not only affects the particle morphology, but can also control the particle size and distribution by changing the system state.

### 3.17. Solubility of Soybean Flour

**Figure 11:**
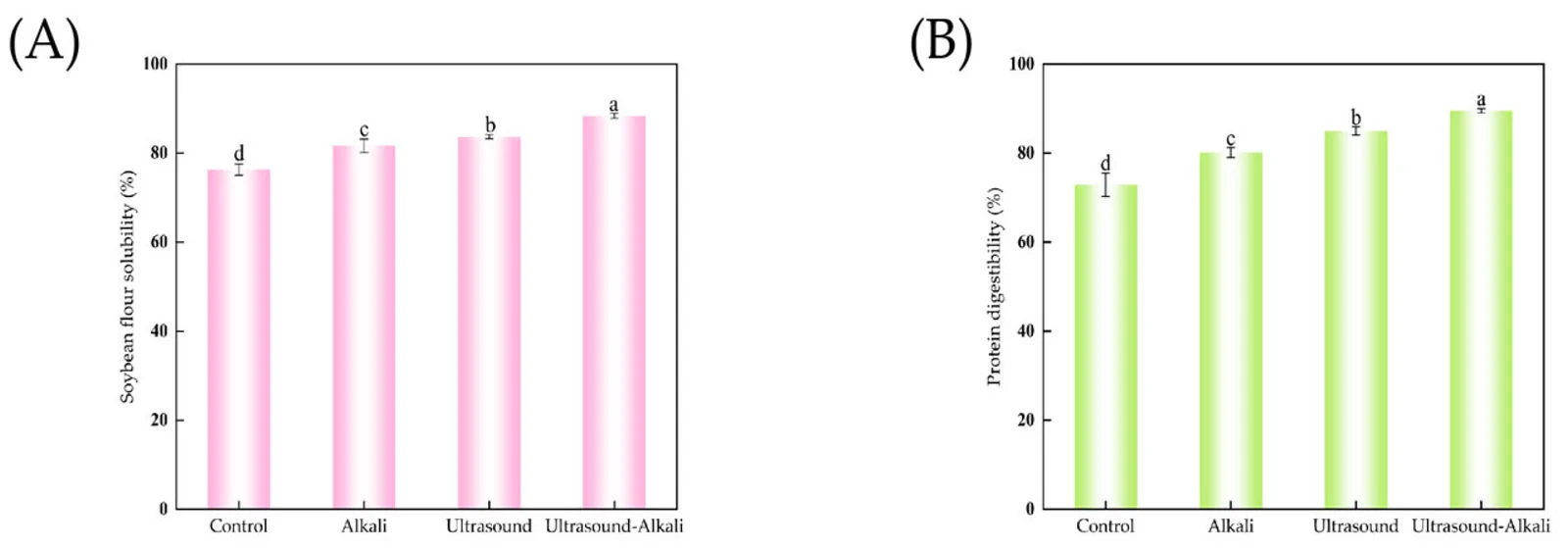
Solubility (**A**) and digestibility (**B**) soybean flour under the different treatments. Different letters indicate significant differences (*p* < 0.05).

Solubility is a key indicator of the quality and rehydration performance of soybean flour. As shown in Figure 11A, compared with the untreated sample, soybean flour showed higher solubility after each treatment, suggesting that processing enhanced its solubility. This change may be related to the increase in the proportion of soluble protein, loose protein structure and exposure of hydrophilic groups, thus enhancing the hydration ability of the powder [75]. Among the different treatment methods, ultrasound combined with alkali treatment improved the solubility of soybean flour most significantly, reaching 88.37% (*p* < 0.05), which was significantly higher than that of single alkali treatment and ultrasound treatment. A possible reason is that the combined treatment reduced particle size, optimized particle distribution, and promoted interaction between the powder and water; at the same time, it promotes the further expansion of protein, exposes more hydrophilic groups, and finally improves the hydration and solubility of soybean flour [76].

### 3.18. Digestibility of Soybean Flour

Protein digestibility is the key index to evaluate the nutritional value of soybean flour and guide the optimization of processing technology [77]. Figure 11B reveals that the digestibility of soybean flour increased after different processing treatments. Compared with the untreated group, the solubility of soybean flour was improved by the various treatments, which may be attributed to treatment-induced changes in the protein structure of soybean flour, leading to protein unfolding, exposure of enzymatic cleavage sites, and a substantial increase in enzyme accessibility, thereby enhancing its digestibility. This result is consistent with the findings of Li Zhiming et al. [78], who reported that the unfolding of protein molecules exposed more enzymatic cleavage sites, which was beneficial to enzymatic hydrolysis. Compared with alkaline treatment alone and ultrasound treatment alone, combined treatment resulted in the highest digestibility of soybean flour, reaching 89.48% (*p* < 0.05). This may be because it reduced the particle size of soybean flour, thereby contributing to improved digestibility. This finding is in agreement with the report of Hu et al. [79]; ultrasound promoted the conversion of proteins into smaller peptides during fibrillation, resulting in the highest protein digestibility.

### 3.19. SDS-PAGE of Soybean Flour After In Vitro Digestion

**Figure 12:**
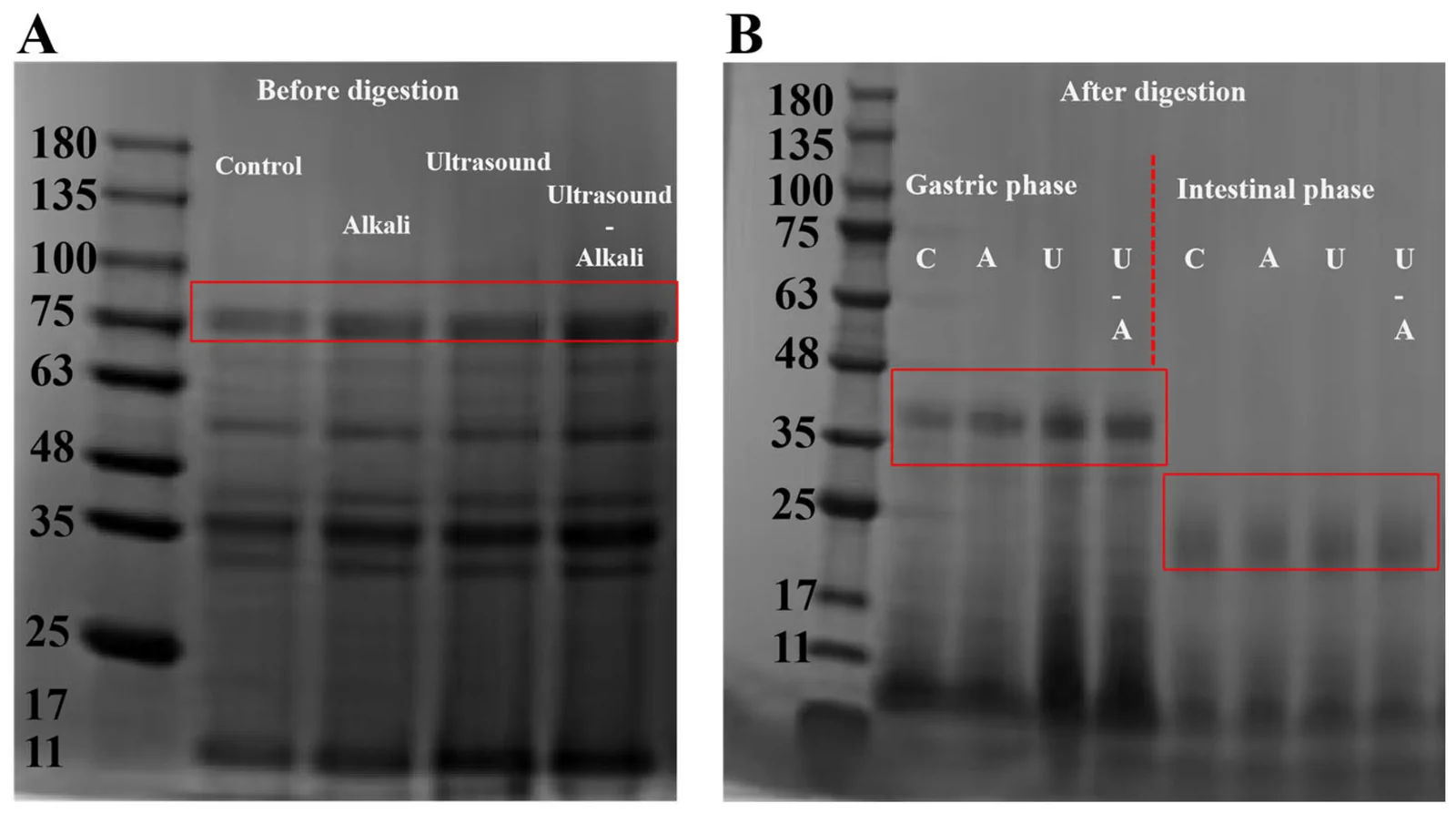
SDS-PAGE profiles of soybean flour before and after in vitro digestion. Before digestion (**A**), After digestion (gastric and intestinal phases) (**B**). Dashed lines separate different digestion stages. Abbreviations: C, Control; A, Alkali; U, Ultrasound; U-A, Ultrasound + Alkali. Molecular weight markers (kDa) are shown on the left.

By measuring the changes in electrophoretogram after in vitro digestion of soybean flour, the changes in molecular weight distribution and subunit composition of protein hydrolysates can be qualitatively analyzed, which provides a molecular basis for revealing the law of protein degradation and nutritional characteristics during digestion. The electrophoresis of different processed soymilk before and after in vitro digestion is shown in Figure 12. Before in vitro digestion, the electrophoresis band of soybean flour treated by ultrasound combined with alkali is the deepest than that of other processed soybean flour, indicating that its protein content is the highest, which is consistent with the increase in soluble protein content [80]. After in vitro digestion, the color of the 35 kDa band decreased significantly, which may be due to the reduction in protein molecular weight caused by pepsin hydrolysis. However, the color of soybean flour strips treated by ultrasound combined with alkali is the darkest, which means that it has the least loss in the stomach. With the further hydrolysis of trypsin, the molecular band of about 35 kDa disappeared, and the color of the band with lower molecular weight deepened, indicating that the macromolecular protein was degraded by trypsin to produce small peptides. At the same time, soybean flour treated with combined ultrasound and alkali treatment showed a lighter color than that of the other treatments, which was consistent with the trend in protein digestibility, indicating that combined ultrasound and alkali treatment improved the digestibility of soybean flour. This may be attributed to the structural unfolding of soy protein induced by combined ultrasound and alkali treatment, which increases its susceptibility to hydrolysis into smaller peptides, thereby enhancing digestibility and absorption.

## 4. Conclusions

In this study, the effects of combined ultrasound and NaHCO_3_ pretreatment on the structure of soymilk proteins, the stability of soymilk, and the physicochemical and digestive properties of soybean flour were systematically investigated. The results showed that protein extraction yield in soymilk was increased to 81.12%, and the protein retained in okara was reduced to 19.74%. At the same time, the average particle size of soymilk decreased from 132.6 μ m to 90.61 m, and the apparent viscosity and viscoelastic modulus were higher than those of untreated samples. In addition, the combined treatment significantly increased the emulsifying activity index (35.49 m^2^/g), emulsion stability and interfacial protein content (62.77%), while reducing the interfacial tension and increasing the dynamic interfacial pressure. The results of Fourier transform infrared spectroscopy, secondary structure analysis, endogenous fluorescence and surface hydrophobicity showed that the structure of soymilk protein expanded, more polar groups were exposed to the water phase, and the molecular flexibility increased, which enhanced the storage stability, ionic stability and freeze–thaw stability of soymilk. After spray-drying, the obtained soybean flour showed smaller and more dispersed microstructure, higher solubility (88.37%) and higher in vitro digestibility (89.48%) than the other samples. Overall, combined ultrasound and NaHCO_3_ pretreatment improved soybean flour quality by enhancing protein solubilization and interfacial functionality.

## Figures and Tables

**Table 1:** Effect of different processing treatments on the secondary structure of soybean milk interfacial protein.

Samples	α−Helix	β−Sheet (%)	β−Turn (%)	Random Coil (%)
Control	25.53 ± 0.51 ^a^	12.53 ± 0.15 ^d^	20.43 ± 0.64 ^d^	41.57 ± 0.31 ^d^
Alkali	23.4 ± 0.3 ^b^	13.9 ± 0.1 ^c^	22.07 ± 0.49 ^c^	42.4 ± 0.26 ^c^
Ultrasound	20.3 ± 0.26 ^c^	14.87 ± 0.5 ^a^	23.4 ± 0.62 ^b^	43.3 ± 0.53 ^b^
Ultrasound–Alkali	18.2 ± 0.4 ^d^	15.37 ± 0.51 ^a^	24.77 ± 0.58 ^a^	44.23 ± 0.45 ^a^

Note: Means with the different superscript letters within the same column for each parameter are significantly different (*p* < 0.05).

## Data Availability

The original contributions presented in the study are included in the article, further inquiries can be directed to the corresponding author.
